# Preload-Based Starling-Like Control for Rotary Blood Pumps: Numerical Comparison with Pulsatility Control and Constant Speed Operation

**DOI:** 10.1371/journal.pone.0121413

**Published:** 2015-04-07

**Authors:** Mahdi Mansouri, Robert F. Salamonsen, Einly Lim, Rini Akmeliawati, Nigel H. Lovell

**Affiliations:** 1 Department of Biomedical Engineering, University of Malaya, Kuala Lumpur, Malaysia; 2 Department of Intensive Care, Alfred Hospital, Prahran, VIC, Australia; 3 Department of Epidemiology and Preventive Medicine, Monash University, Melbourne, VIC, Australia; 4 Department of Mechatronics Engineering, International Islamic University Malaysia, Kuala Lumpur, Malaysia; 5 Graduate School of Biomedical Engineering, University of New South Wales, Sydney, NSW, Australia; University of Adelaide, AUSTRALIA

## Abstract

In this study, we evaluate a preload-based Starling-like controller for implantable rotary blood pumps (IRBPs) using left ventricular end-diastolic pressure (PLVED) as the feedback variable. Simulations are conducted using a validated mathematical model. The controller emulates the response of the natural left ventricle (LV) to changes in PLVED. We report the performance of the preload-based Starling-like controller in comparison with our recently designed pulsatility controller and constant speed operation. In handling the transition from a baseline state to test states, which include vigorous exercise, blood loss and a major reduction in the LV contractility (LVC), the preload controller outperformed pulsatility control and constant speed operation in all three test scenarios. In exercise, preload-control achieved an increase of 54% in mean pump flow ($\overset{-}{Q_{P}}$) with minimum loading on the LV, while pulsatility control achieved only a 5% increase in flow and a decrease in mean pump speed. In a hemorrhage scenario, the preload control maintained the greatest safety margin against LV suction. PLVED for the preload controller was 4.9 mmHg, compared with 0.4 mmHg for the pulsatility controller and 0.2 mmHg for the constant speed mode. This was associated with an adequate mean arterial pressure (MAP) of 84 mmHg. In transition to low LVC, $\overset{-}{Q_{P}}$ for preload control remained constant at 5.22 L/min with a PLVED of 8.0 mmHg. With regards to pulsatility control, $\overset{-}{Q_{P}}$ fell to the nonviable level of 2.4 L/min with an associated PLVED of 16 mmHg and a MAP of 55 mmHg. Consequently, pulsatility control was deemed inferior to constant speed mode with a PLVED of 11 mmHg and a $\overset{-}{Q_{P}}$ of 5.13 L/min in low LVC scenario. We conclude that pulsatility control imposes a danger to the patient in the severely reduced LVC scenario, which can be overcome by using a preload-based Starling-like control approach.

## Introduction

Preload sensitivity of the ventricular myocardium is an essential requirement for the Frank-Starling mechanism by which the left ventricular end-diastolic pressure (PLVED) controls the force of contraction of the left ventricle (LV) in proportion to the blood flow received from the right heart and pulmonary circulation. Unfortunately, implantable rotary blood pumps (IRBPs) which are currently the preferred technology for assisting the failing LV do not have sufficient preload sensitivity to perform this task automatically [1]. It would seem logical therefore that LV preload be selected as the feedback variable of choice in physiological control systems designed for IRBPs. However, this has not happened seemingly because LV preload is not easily estimated nor measured. In a comprehensive review conducted recently, AlOmari et al. [2] reported few instances of physiological control based on invasive pressure measurements [3–6].

On the other hand, there is a widespread view that currently available implantable pressure transducers are rendered virtually unusable due to a range of problems. These include limited reliability, drifts in transducers’ response over time, and the anatomical distortion they present to pump inlet cannulae, resulting in unwanted flow turbulence and associated clotting disorders. As an alternative, there has been much interest in non-invasive estimation of pump pulsatility measures derived from various pump parameters, either as a display to aid manual adjustment of pump speed [7–10], or as a feedback variable for physiological controllers [11–15]. The theoretical link between pulsatility (the difference between maximum and minimum points on a waveform measured over a cardiac cycle) and PLVED is via the effect of PLVED on the left ventricular stroke work, which in turn affects pulsatility. Pulsatility measures reported include pump flow pulsatility (PIQp), pressure head pulsatility, speed pulsatility and motor current pulsatility.

New technology in blood pressure measurement however brings with it the promise of greater biocompatibility and stability over time. In a multicenter study, Troughton et al. reported that in the Heart Pod pressure transducer, after an initial ‘bedding in’ process in the left atrium, the pressure response was essentially stable over the study period of four years [16]. In addition, they reported an overall 95% freedom from failure over two years, 88% over four years and 100% freedom from failure in the last 41 consecutive patients. More recently, pressure transducers based on optical fibers have been described [17,18], which may achieve the required stability in the constant temperature environment of the heart and are small enough to be embedded in the walls of the pump inlet and outlet cannulae without anatomical distortion.

It is therefore of interest to examine the performance of preload control in comparison with PIQp control and constant speed operation (prevalent in the majority of IRBPs currently implanted clinically). If preload control was shown to be functionally superior to both PIQp and constant speed modes, this might provide the impetus for further development of preload-based Starling-like control. We report such a study using a sophisticated and experimentally validated computer model of the human circulation and the VentrAssist left ventricular assist device (LVAD).

## Methods

### Description of the Heart-Pump Interaction Model

The heart-pump interaction model used in the present study has recently been developed and validated to investigate the response of IRBP-assisted patients to exercise and head up tilt [19]. The basic structure of the model consists of the left and right sides of the heart, the pulmonary and systemic circulations, as well as the LVAD. The LVAD component includes the description of the VentrAssist LVAD operating between 1600 and 3000 rpm, as well as the inlet and outlet cannulae. Furthermore, the model takes into account various important items such as the arterial and cardiopulmonary reflexes, local metabolic vasodilatation in the active muscles, the auto-regulation mechanism in the lower body, as well as the muscle pump. The model was implemented using the SIMULINK toolbox in MATLAB (The Math Works, Inc., Natick, MA, USA). A detailed description of the model validation procedures together with the optimized model parameter values can be obtained from [19–23].

### Description of the Control Systems

#### a. Preload Based Starling-Like Controller

The immediate response of the preload controller emulates the Frank-Starling control mechanism of the natural heart, which was first identified by Starling and Visscher [24] as a sigmoid relationship between LV stroke work and PLVED. This was subsequently modified by Guyton [25] to give a similar sigmoid relationship between LV flow and PLVED.

**Fig 1:**
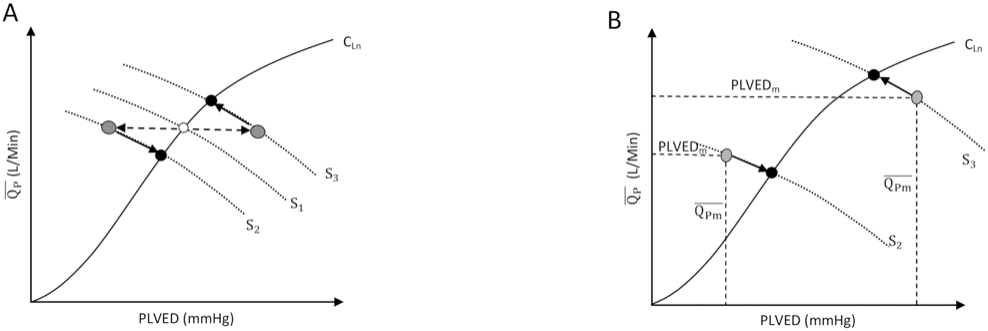
Schematic describing the preload-based Starling-like control, A) Frank-starling curve, B) returning the operating point back to C_L_. C_L_, Control line of the pump (Frank-Starling curve); S_1_, original system state corresponding to the original left ventricular end-diastolic pressure (PLVED); S_2_ and S_3_, changed system states. PLVED, Left Ventricular End Diastolic Pressure; $\overset{-}{Q_{P}}$, mean pump flow; PLVED_m_, measured (actual) left ventricular end diastolic pressure provided by the model; $\overset{-}{Q_{Pm}}$, measured (actual) mean pump flow provided by the model; White circle, position of operating point (current combination of PLVED and $\overset{-}{Q_{P}}$) before a change of state; Grey circles, position of operating points after changes in states; Black circles, position of operating points upon arriving at the new steady state located at the intersection between the control line and the new system line. The controller drives the changes in the operating points along the path indicated by the arrows along the new system line.

The Frank-Starling curve forms the basis of the control line (C_L_), as illustrated in Fig. 1A. In the proposed preload controller, C_L_ is generated by a third order polynomial function (1) fitted directly to Guyton’s data [25], which relates desired mean pump flow ($\overset{-}{Q_{P}})$ to PLVED. A scaling factor (K) is also added to provide a means of altering sensitivity of the pump to changes in PLVED, which makes (1) adaptive with different patients’ preload sensitivity [26, 27].

$$\bar{{\text{Q}}_{\text{P}}}=\left(0.0003{*PLVED}_{\text{m}}^{3}-0.0276{*PLVED}_{\text{m}}^{2}+0.9315{*PLVED}_{\text{m}}-0.0928\right)*\text{K}$$ (1)

where PLVED_m_ represents the LV end diastolic pressure provided by the numerical cardiovascular (CVS) model. In our study, PLVED_m_ is automatically sampled at end diastole of each heart cycle by the model and then fed to the controller. Although the full controller is able to adapt to longer term changes in the circulation by adjustment of the scaling factor (K), this communication deals only with the immediate response of the controller in which changes in PLVED cause migration of the operating point to different positions on C_L_.

Fig. 1A gives an overall view of how the preload controller functions in a diagrammatic form. As changes in state of the subject evolve (transition from state S_1_ to states S_2_ or S_3_), these changes are tracked by the controller which then returns the operating point back to the control line (C_L_). For example, the white circle in Fig. 1A gives the position of the original operating point, while the grey circles give its position after a deviation from the control line, induced by changes in the system state from *S*_1_ to *S*_2_ or *S*_3_. Fig. 1B presents details on how the controller returns the operating point back to C_L_ along a linear path in a series of steps (indicated by the small arrows), until it settles to a new position located at the intersections between C_L_ and the altered system states (indicated by black circles). Thus, deviations of the operating point from C_L_ either to the right or left side of the control line move the operating point upwards or downwards along C_L_.

#### b. Pulsatility Controller

The pulsatility controller, on the other hand, relates the desired mean pump flow ($\overset{-}{Q_{P}})$ with pump flow pulsatility (PIQ_Pm_), defined as the absolute difference between the maximum and the minimum pump flow [26] over a heartbeat. Instead of being curvilinear, the control line for the pulsatility controller is linear (2) as derived by Salamonsen et al., with the gradient defined by the tangent of the angle (θ) it makes with the pulsatility axis in radians. Similar to preload control, reference changes are applied by movements of the operating point up or down C_L_. However, since C_L_ is linear, the controller is able to force the deviated operating point back to the appropriate position on C_L_ along a circular path, as defined by (2) [15].

$$\bar{{\text{Q}}_{\text{P}}}=\left(\sqrt{{\left(\bar{{\text{Q}}_{\text{Pm}}}\right)}^{2}+{\left({\text{PiQ}}_{\text{Pm}}\right)}^{2}}\right)*\sin(\theta )$$ (2)

where $\overset{-}{Q_{P}}$ represents the desired average pump flow, $\overset{-}{Q_{Pm}}$ is the mean pump flow provided by the model, and PIQ_Pm_ is the pump flow pulsatility. More detailed descriptions of the pulsatility controller can be obtained from [15] and [12].

### Simulation Protocols

**Fig 2:**
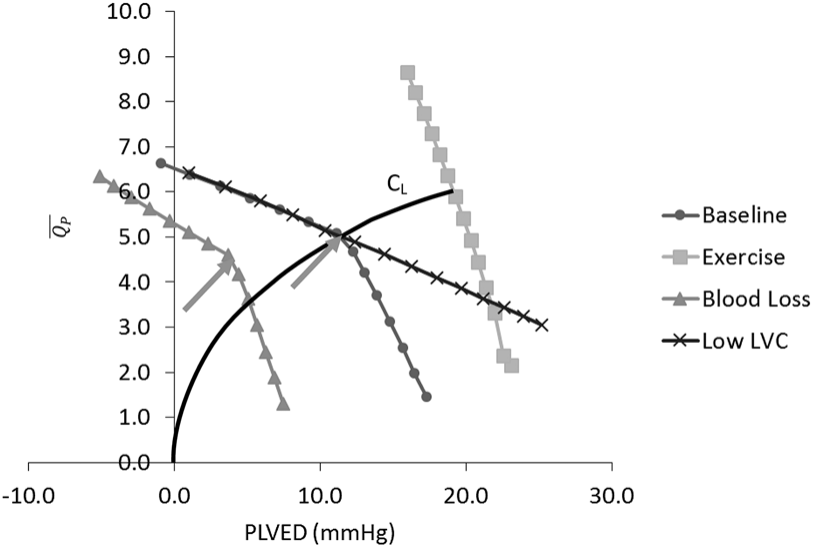
System response to variations in mean pump flow ($\overset{-}{Q_{P}}$) for baseline and three test conditions. Fig. also shows the superimposed control line (C_L_), where the minimum scaling factor (K) that allows the aortic valve to be closed in the baseline condition was chosen. LVC, left ventricular contractility; PLVED, left ventricular end-diastolic pressure; $\overset{-}{Q_{p}}$, mean pump flow; Arrows indicate points where the aortic valve starts to open.

In order to determine the gradient for the return path to the control line, model simulations were performed for the baseline, exercise, blood loss and reduced LV contractility conditions, in which mean pump speed ($\overset{-}{\omega_{P}}$) was increased from 1600 rpm to 3000 rpm in 100 rpm increments. Mean flow through the aortic valve ($\overset{-}{Q_{av}}$) and pump ($\overset{-}{Q_{P}}$) as well as PLVED were obtained from the model, with the relationship between $\overset{-}{Q_{P}}$ and PLVED plotted in Fig. 2. System gradients with AV open and AV closed were calculated separately for each test scenario, and the mean value for each AV state was then selected as the gradients of the return path.

It can be shown from Table 1 that although system lines for the different test scenarios showed wide displacements among each other, their gradients grouped according to whether the aortic valve was opening or closing were similar.

**Table 1:** Gradients for return lines (ratio of mean pump flow to PLVED) for baseline and three test scenarios, i.e. exercise, blood loss and reduced left ventricular (LV) contractility scenario.

System States	Gradient with AV Open (L/min/mmHg)	Gradient with AV Closed (L/min/mmHg)
Baseline	-0.64	-0.13
Exercise	-0.92	---
Blood Loss	-0.94	-0.19
Low LVC	---	-0.14
Mean	-0.83	-0.15

During exercise, the aortic valve (AV) remained open throughout the range of speed tested, while in reduced LV contractility (LVC) scenario (Low LVC), the AV remained closed.

In normal clinical practice, RBP speed is set to stop the aortic valve from opening in order to provide full assist to the patients [28]. The aortic valve opening condition is only used while attempting to wean a patient from the pump or used intermittently to prevent inappropriate sealing of the aortic valve cups. In order to determine the optimal scaling factor (K) for C_L_, another set of simulations was conducted in the baseline state, where K was increased from a basal value of 0.2 to the maximal value of 2.3 in 0.1 increments. Blood flow through the aortic valve ($\overset{-}{Q_{av}}$) was obtained from the model to indicate whether the aortic valve was opening or closing. The optimal scaling factor was identified as the minimum value of K that allowed the aortic valve to be closed in the baseline condition. In the present study, K value was set to 1.0, while the corresponding control line of the pulsatility controller had a gradient (angle), θ of 62^0^.

**Fig 3:**
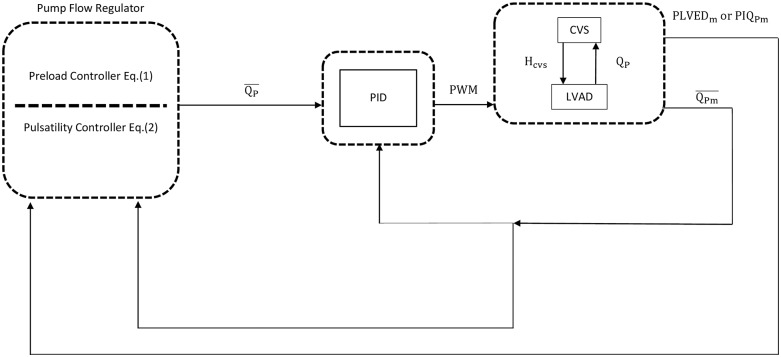
Block diagram of the PID controller for closed loop studies. CVS, cardiovascular system; LVAD, left ventricular assist device; PWM, pulse width modulation; PID, Proportional-integral-derivative controller; Q_P,_ instantaneous pump flow; $\overset{-}{Q_{P}}$, mean pump flow; $\overset{-}{Q_{Pm}}$, measured mean pump flow; PLVED_m_, measured left ventricular end diastolic pressure; PIQ_Pm,_ measured pump flow pulsatility; H_CVS_, differential pressure between the left ventricle and the aorta. PLVED_m_ serves as the input to the preload controller (1), while both PIQ_Pm_ and $\overset{-}{Q_{Pm}}$ are inputs to the pulsatility controller (2).

Both pulsatility and preload-based Starling-like control modes were then implemented separately with a proportional-integral-derivative (PID) controller [29] (Fig. 3), which adjusted the pulse-width-modulation (PWM) signal to generate the required $\overset{-}{Q_{P}}$. The transfer function of the PID controller is defined in (3), and was discretized automatically by MATLAB/SIMULINK using a sampling period of 0.002 seconds.

$$\text{G}\left(\text{S}\right)={\text{K}}_{\text{P}}+\frac{{\text{K}}_{\text{I}}}{\text{S}}+{\text{K}}_{\text{D}}\text{S}$$ (3)

K_P_, K_I_ and K_D_ represent the proportional, integral and derivative gains of the controller, respectively (Table 2). The values of these constant gains were tuned to achieve a 5% settling time of 10 seconds with minimal overshoot (i.e. a maximum overshoot within 10% of the final value). In each iteration, the PID controller compared the mean pump flow ($\overset{-}{Q_{Pm}}$) obtained from the model with the desired mean pump flow ($\overset{-}{Q_{P}}$), and moved the operating point back to the control line following the selected return path.

**Table 2:** Proportional-Integral-Derivative (PID) gains used for both preload and pulsatility controlling methods.

Control constants	Gains
K_P_	0.28
K_I_	0.22
K_D_	0.05

K_P_, Proportional gain; K_I_, Integral Gain; K_D_, Derivative Gain.

In addition to the baseline state at rest, three other scenarios, including exercise, blood loss and reduced LV contractility were simulated. For the exercise simulation, a relative intensity of 0.55 was chosen to represent the maximal exercise condition as defined by Lim et al. [19]. To simulate blood loss and reduced LV contractility conditions, total blood volume (V_total_) and maximum LV end systolic elastance (E_max,lv_) were reduced by 1000 mL and 78%, respectively. The steady-state performance of both preload and pulsatility control were compared to each other as well as with the constant speed mode. For all simulations, we waited for the hemodynamic variables to settle to a steady state condition before transitioning from the baseline into one of the three test conditions. The simulation was then continued for a sufficient period to allow the control modes to achieve a post transition steady state.

## Results

**Fig 4:**
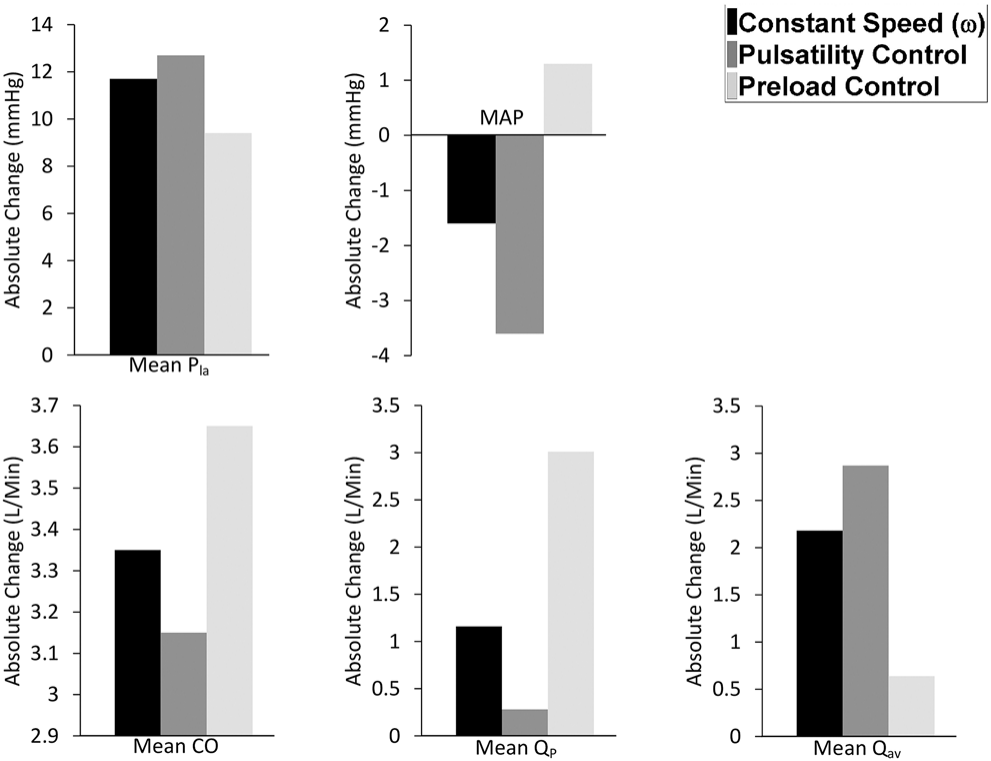
Comparison of preload controller vs. pulsatility and constant speed modes from baseline to exercise. Absolute value changes in the: P_la_, left atrial pressure; MAP, mean arterial pressure; CO, cardiac output; Q_P_, pump flow; Q_av_, aortic valve flow.

**Fig 5:**
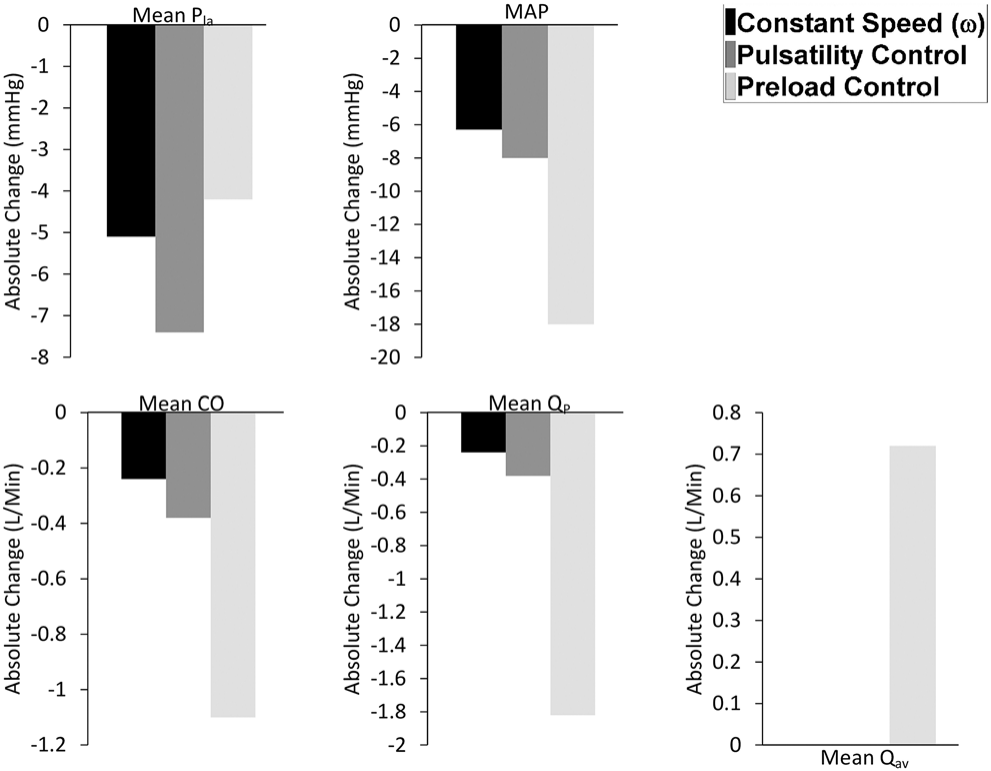
Comparison of preload controller vs. pulsatility and constant speed modes from baseline to hemorrhage. Absolute value changes in the: P_la_, left atrial pressure; MAP, mean arterial pressure; CO, cardiac output; Q_P_, pump flow; Q_av_, aortic valve flow.

**Fig 6:**
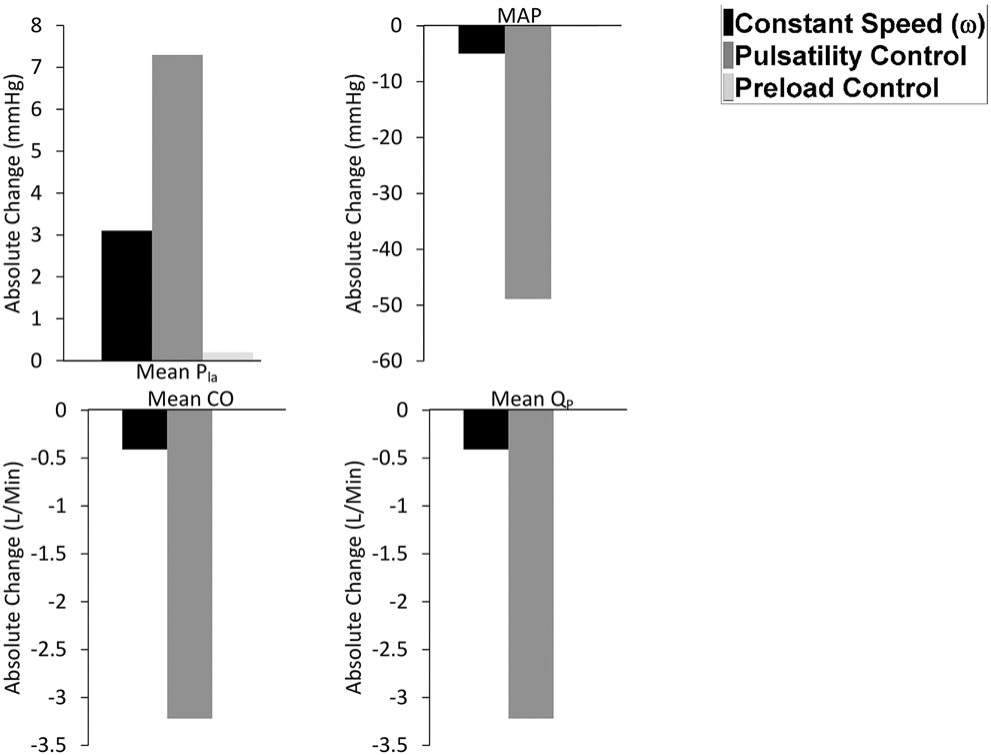
Comparison of preload controller vs. pulsatility and constant speed modes from baseline to reduced left ventricular contractility scenario. Absolute value changes in the: P_la_, left atrial pressure; MAP, mean arterial pressure; CO, cardiac output; Q_P_, pump flow.

The performance of the three control methods was compared by observing the changes in mean pump flow ($\overset{-}{Q_{P}}$), mean cardiac output ($\overset{-}{CO}$), mean aortic valve flow ($\overset{-}{Q_{av}}$), mean systemic arterial pressure (MAP), and mean left atrial pressure ($\overset{-}{P_{la}}$) from baseline to exercise (Fig. 4), hemorrhage (Fig. 5) and reduced left cardiac contractility (Fig. 6) scenario.

Results indicate that during exercise, preload control was the best controlling modality, causing a 55% increase in $\overset{-}{Q_{P}}$ and a 66% increase in $\overset{-}{CO}$. This was associated with the lowest values for both PLVED (16.1 mmHg) and $\overset{-}{Q_{av}}$ (0.64 L/min) among the three controllers, indicating minimum load on the LV, as depicted in Table 3 and Fig. 4. Pulsatility control gave the poorest performance among the three control modes, with a mere increase of 5% in $\overset{-}{Q_{P}}$, associated with a fall in $\overset{-}{\omega_{P}}$. This was due to an increase of 21% in $\overset{-}{Q_{P}}$ at constant speed ($\overset{-}{\omega_{P}}$ = 2570 rpm), to which pulsatility was very sensitive, and the resultant failure of PIQp to rise in proportion to the degree of exercise. This results in an increase in the LV stroke work index. Consequently, pulsatility control was associated with the highest $\overset{-}{Q_{av}}$ (2.87 L/min) during exercise and the maximum increase in PLVED (168% rise).

**Table 3:** Model simulated hemodynamic data at baseline (rest) and exercise with different controllers.

Variable	Unit	Control Speed		Pulsatility Control		Preload Control	
		Rest	Exercise	Rest	Exercise	Rest	Exercise
$\overset{-}{\omega_{P}}$	RPM	2570	2570	2585	2410	2570	2980
MAP	mmHg	102.4	100.8	103.4	99.8	102.4	103.7
$\overset{-}{P_{la}}$	mmHg	9.5	21.2	8.9	21.6	9.6	19.6
PLVED	mmHg	7.8	18.7	7.2	19.3	8.0	16.1
$\overset{-}{CO}$	L/min	5.54	8.89	5.62	8.77	5.52	9.17
$\overset{-}{Q_{P}}$	L/min	5.54	6.70	5.62	5.90	5.52	8.53
PIQp	L/min	2.90	2.90	3.07	3.28	3.05	2.68
$\overset{-}{Q_{av}}$	L/min	0.00	2.18	0.00	2.87	0.00	0.64

$\overset{-}{\omega_{P}}$, mean rotational speed; MAP, mean arterial pressure; $\overset{-}{P_{la}}$, mean left atrial pressure; PLVED, left ventricular end-diastolic pressure; $\overset{-}{CO}$, mean cardiac output; $\overset{-}{Q_{P}}$, mean pump flow; PIQp, pump flow pulsatility; $\overset{-}{Q_{av}}$, mean aortic valve flow.

The challenge for an LVAD during a blood loss scenario is to avoid LV suction by reducing its flow output sufficiently to match a substantial reduction in the right ventricular (RV) preload and subsequent reduction in the delivery from the RV. In our model simulation, LV suction was indicated by a negative PLVED (i.e. PLVED ≤ 0). As observed in our previous animal experimental studies [30], LV suction involves obstruction of the pump inlet cannula due to suction of the LV walls at relatively high pump speeds. In this state, LV volume is low, resulting in steady near zero LV pressure and negative pump inlet pressure throughout the cardiac cycle. Our observations were consistent with Karantonis et al. [31] and Boston et al. [32], who suggested that LV suction caused a negative pressure in the LV, and thus it is imperative to maintain left atrial pressure (substitute of PLVED) above 0 mmHg to avoid LV suction. Based on this suction indicator and results in Table 4 and Fig. 5, preload control was the only modality that was able to reduce flow sufficiently to maintain an adequate safety margin against LV suction. PLVED was 4.9 mmHg for preload control after a reduction in the total blood volume, in contrast to near zero PLVEDs for constant speed mode (0.2 mmHg) and pulsatility control mode (0.4 mmHg). Baroreceptor reflexes, which the model was equipped with, were sufficient to avoid abrupt falls in the mean arterial pressure (MAP), even though this particular parameter was not specifically addressed in the controlling policy. MAP was reduced from 102 mmHg to 84 mmHg upon blood loss, which is more than sufficient to maintain auto-regulation of flow by the body tissues (minimum threshold = 60 mmHg) [33].

**Table 4:** Model simulated hemodynamic data at baseline (rest) and with blood-loss for different controllers.

Variable	Unit	Control Speed		Pulsatility Control		Preload Control	
		Rest	Exercise	Rest	Exercise	Rest	Exercise
$\overset{-}{\omega_{P}}$	RPM	2570	2570	2585	2550	2570	2115
MAP	mmHg	102.4	96.1	103.4	95.4	102.4	84.4
$\overset{-}{P_{la}}$	mmHg	9.5	4.4	8.9	1.5	9.6	5.4
PLVED	mmHg	7.8	0.2	7.2	0.4	8.0	4.9
$\overset{-}{CO}$	L/min	5.54	5.30	5.62	5.24	5.52	4.42
$\overset{-}{Q_{P}}$	L/min	5.54	5.30	5.62	5.24	5.52	3.7
PIQp	L/min	2.90	2.54	3.07	2.87	3.05	4.06
$\overset{-}{Q_{av}}$	L/min	0.00	0.00	0.00	0.00	0.00	0.72

$\overset{-}{\omega_{P}}$, mean rotational speed;MAP, mean arterial pressure; $\overset{-}{P_{la}}$, mean left atrial pressure; PLEVD, left ventricular end-diastolic pressure; $\overset{-}{CO}$, mean cardiac output; $\overset{-}{Q_{P}}$, mean pump flow; PIQp, pump flow pulsatility; $\overset{-}{Q_{av}}$, mean aortic valve flow.

Preload control was able to maintain an adequate $\overset{-}{CO}$ despite a reduction in LV contractility, where $\overset{-}{Q_{P}}$ and MAP remained constant at 5.52 L/min and at 102 mmHg respectively, without a major rise in PLVED (Table 5 and Fig. 6). To the contrary, with pulsatility control, $\overset{-}{Q_{P}}$ (equivalent to $\overset{-}{CO}$ as aortic valve was closed) fell to the non-viable level of 2.4 L/min and MAP to 51 mmHg. In doing so, it was less supportive to the circulation than the constant speed mode which maintained an average pump flow of 5.13 L/min and MAP of 97 mmHg.

**Table 5:** Model simulated hemodynamic data at baseline (rest) and fall in left ventricular contractility.

Variable	Unit	Control Speed		Pulsatility Control		Preload Control	
		Rest	Exercise	Rest	Exercise	Rest	Exercise
$\overset{-}{\omega_{P}}$	RPM	2570	2570	2585	1575	2570	2700
MAP	mmHg	102.4	97.4	103.4	54.5	102.4	102.2
$\overset{-}{P_{la}}$	mmHg	9.5	12.6	8.9	16.2	9.6	9.8
PLVED	mmHg	7.8	11.0	7.2	15.7	8.0	8.0
$\overset{-}{CO}$	L/min	5.54	5.13	5.62	2.40	5.52	5.52
$\overset{-}{Q_{P}}$	L/min	5.54	5.13	5.62	2.40	5.52	5.52
PIQp	L/min	2.90	0.52	3.07	0.80	3.05	0.57
$\overset{-}{Q_{av}}$	L/min	0.00	0.00	0.00	0.00	0.00	0.00

$\overset{-}{\omega_{P}}$, mean rotational speed; MAP, mean arterial pressure; $\overset{-}{P_{la}}$, mean left atrial pressure; PLEVD, left ventricular end-diastolic pressure; $\overset{-}{CO}$, mean cardiac output; $\overset{-}{Q_{P}}$, mean pump flow; PIQp, pump flow pulsatility; $\overset{-}{Q_{av}}$, mean aortic valve flow; LVC, Left ventricular contractility.

## Discussion

This numerical simulation study clearly establishes the utility of a single preload-based Starling-like control line to control $\overset{-}{Q_{P}}$ appropriately in the transition from rest to three simulated scenarios: vigorous exercise, severe blood loss and a major fall in LV contractility. It consistently outperformed the pulsatility controller, whose performance was inferior even to the fixed speed mode. These results agree with a recent study reported by Lim et al. [19], which highlighted the deficiencies of the pulsatility control in exercise and 70 degree head up tilt.

### Physiological Mechanisms

On a theoretical basis, there are sound physiological mechanisms underlying these results. Pump pulsatility (flow, current, pressure gradient or speed) is a consequence of LV contraction whereas LV preload is one of the determinants of LV contraction. With severe LV failure, as is the case for all LVAD recipients, the LV does not have the ability to induce major changes in pulsatility. Therefore, the dynamic range of the pulsatility index is small, and as a consequence its ability as a control input is limited. In the extreme case where LV contractility is zero, pulsatility control is not feasible. By contrast, LV preload increases as LV failure progresses, and its dynamic range is wide in the presence of LVAD.

These fundamental characteristics account for the superiority of the preload control in all three test simulations. In addition, other mechanisms come into play with each of the three test states. With exercise, the natural increase in $\overset{-}{Q_{P}}$ during exercise at constant speed severely reduces PIQp. Consequently, when the operating point is returned to the control line in pulsatility control, there is an actual decrease in mean pump speed as reported by Salamonsen, et al. 2013 [34]. Preload is much less sensitive to this effect and progressively rises with increasing exercise intensity, thus accounting for the observed superiority of preload control.

In blood loss, because of the low dynamic range of the pulsatility index, the ability of the pulsatility control to reduce $\overset{-}{Q_{P}}$ effectively is limited and thus the risk of LV suction is greater. Another major problem with pulsatility control is that PIQp, being a consequence of the LV stroke work, is unable to distinguish between blood volume loss and a fall in LV contractility since PIQp falls in both occasions. This is a major disadvantage as the role of the LVAD in the two conditions should be very different. By contrast, preload control responds to both conditions effectively, with a loss of blood volume causing a reduction in $\overset{-}{Q_{P}}$ to avoid LV suction while a reduction in LV contractility causing an opposite effect to provide sufficient flow to the systemic circulation.

By emulating the Frank-Starling control mechanism of the natural heart, preload-based Starling-like control is able to synchronize LV and right ventricular (RV) outputs irrespective of variations in venous return [15]. Compared to constant pulsatility and constant speed modes (Table 3 & 5), preload control produced the least increase in PLVED during exercise and reduced LV contractility scenarios, thus reducing the chances of pulmonary congestion which may lead to right-sided circulatory failure in the long term [35]. In addition, preload control was able to maintain an adequate safety margin against LV suction with a reduction in the total blood volume. LV suction may cause a significant reduction in the right ventricular performance through endocardial damage and septal shift [15]. Depending on the status of the pulmonary vascular resistance, right ventricular contractility and volume status, sustained suction-induced hemodynamic collapse lasting for more than 15 min may occur in serious circumstances, causing unfavorable conditions for effective LVAD unloading [36].

### Nature of Preload-Based Starling-Like Control

To date, most control methods are based on a fixed set point, such as constant speed [37], preload [3], differential pressure [38] or pulsatility [39–40]. Although Bullister et al. [3] also made use of PLVED as their main input variable, they adopted a completely different approach from the preload-based Starling-like controller proposed in the present study. In Bullister’s method, a set point for PLVED which lies within a physician-programmable range was chosen based on the desired range for mean arterial pressure (MAP). Apart from this, a Level 2 control algorithm was activated when the heart rate increased above a resting threshold value, which continuously modified PLVED to achieve the new target value for MAP determined based on the measured heart rate. There are several limitations associated with Bullister’s method. As demonstrated in previous clinical [34,41–42] and simulation studies [19], the level of resting PLVED varies significantly among individuals (5–16.7 mmHg [34,41–42]), and in the face of various physiological perturbations in the circulatory system (5–9 mmHg during exercise [34, 41–42] and -4.5 mmHg during 70^0^HUT [19]). Maintaining PLVED at a fixed set point in the presence of various physiological perturbations, therefore, would require excessive variation in the pump speed. We have shown from our previous simulation studies that constant left atrial pressure control (equivalent to constant PLVED) caused a drastic fall in MAP upon 70^0^HUT [19], which may lead to cases of orthostatic hypertension and subsequently affect circulatory stability. Although Bullister et al. attempted to maintain the MAP within a physician-programmable range, this control loop is reacting much slower, and thus may not be able to cater for sudden circulatory stability caused by abrupt changes in the mean pump speed. Furthermore, heart rate dependency was built into Bullister et al.’s algorithm to increase MAP during exercise, in an attempt to further increase cardiac output. As shown from our simulation results which integrated the reflex mechanism and previous clinical findings [34, 41–42], the level of MAP is mostly determined by the circulatory system (e.g. through a change in the venous unstressed volume and systemic vascular resistance) as well as the sensitivity of the reflex mechanism. In addition, especially in heart failure patients, increasing venous return produced a substantial increase in PLVED during exercise, and in most cases more than the amount of increase in MAP. In view of this, building a MAP-heart rate dependency into the model is redundant in most circumstances, as this may actually slow down the response of the controller to a change in PLVED due to the slower reacting MAP control loop.

To the contrary, our preload-based Starling like controller emulates the Frank-Starling control mechanism of the natural heart, which regulates stroke volume in proportion to the level of venous return (reflected by PLVED) only. Instead of fixing a set point for PLVED which is expected to vary significantly with different physiological perturbations, we regulated mean pump flow in accordance to the measured PLVED using a predefined control line (1), which indicates the state of the circulation and the degree to which it is meeting the physiological requirements of the body at each instant. Apart from that, as described in Section 2.2, preload sensitivity for individual patients could be modified by changing the scaling factor (K in (1)) which provides a means of altering sensitivity of the pump to changes in PLVED. In addition, we did not induce an explicit MAP control, but instead relies on the regulation of MAP by the baroreceptors and other circulatory reflexes [33]. Consequently preload controller is more robust in providing the appropriate level of blood flow to the systemic circulation under various clinical circumstances [19].

This study also indicates that due to its unique shape, a single preload control line (Frank-Starling curve) is able to provide a major decrease in flow at low LV preloads to avoid LV suction and to limit increases at high preloads to avoid over-pumping. Although this study evaluates a single control line, the full controller is able to adapt to longer term changes in the LV function and circulation by adjustment of the scaling factor for the control line to yield different Frank-Starling curves. Having different curves with varying gradients not only provides inherent protection against LV suction, but also determines the degree of LV unloading. Consequently, apart from controlling the level of cardiac output, the preload controller would be able to determine the amount of work performed by the ventricle. Particularly, in the early postoperative phase, it is important that the scaling factor of the control line be selected and modified accordingly by the attending medical staff based on additional clinical requirements, besides the provision of adequate blood flow to the tissues. Later after implantation, the use of upper and lower limits for PLVED and $\overset{-}{Q_{P}}$ would enable the controller to adapt the degree of pump assistance automatically by modifying the scaling factor of the control line [15]. It is noteworthy that the upper and lower limits for PLVED and $\overset{-}{Q_{P}}$ can be adjusted by the clinicians to accommodate for changes in the patients’ condition over time.

### Deficiencies of the Preload-Based Starling-Like Control

The Frank-Starling mechanism in the native heart, being a property of the myocardium, effectively eliminates complications like LV suction, as the ventricle does not pump when it is empty. In addition, the adjustment of the contractile force following an increase in the myocyte length is virtually immediate, being mediated by an adjustment of the number of myofilament cross bridges that interact, as well as by an alteration in the calcium sensitivity of the myofilaments [33]. In order to avoid suction in the presence of an IRBP with low preload sensitivity, the controller must be able to implement reference changes in $\overset{-}{Q_{P}}$ as soon as a change in PLVED is sensed. This is a challenge to most control methods particularly if they require an estimation of $\overset{-}{Q_{P}}$ or pressure head across the pump. While most published methods took two to three heart beats to estimate the average values of the flow [2], only one report estimated instantaneous flow in a pulsatile environment [43]. Similarly, estimation of PLVED presents difficulties, with only one report existing for non-invasive estimation of the mean diastolic pressure which is closely related to PLVED [44]. Therefore, PLVED measurement by pressure transducers is clearly superior because the response is instantaneous.

The approach described herein involves direct LV preload adjustment, but does not induce an explicit MAP control. In contrast to a previous study which implemented MAP control [3], the preload controller relies on the regulation of MAP by the baroreceptors and other circulatory reflexes [33]. Attending medical staffs usually provide additional pharmacological control of systemic blood pressure if required. This usually takes the form of vasodilators rather than vasoconstrictors due to the over action of the sympathetic nervous system in response to the low cardiac output seen in most heart failure patients.

### Inadequacies of the Study and Future Work

The model used in this study has been well validated against animal studies conducted by our group and reported in the scientific literature [19, 22]. In our study, the PLVED was obtained from the model at end diastole of each heartbeat. However, in reality, it may need to be averaged over two or three heart beats due to the presence of measurement noise [45], especially if there are abnormalities in the cardiac rhythm. Similarly, new values for $\overset{-}{Q_{P}}$ will also take at least one or two heart beats to be measured. It thus remains to be tested if the controller is able to adjust $\overset{-}{Q_{P}}$ quickly enough to avoid suction during changes in posture, protracted abdominal straining or coughing in the implanted subject, where the fall in PLVED may be rapid. These points will be addressed in future work.

## Conclusion

This study establishes the clear superiority of the preload control over both constant speed and pulsatility control modes. It provides safe and effective adjustments of $\overset{-}{Q_{P}}$ despite the widely varying states of exercise, blood loss and a fall in LV contractility. This provides impetus for continued efforts to develop miniaturized pressure transducers that are stable over time and small enough to be embedded into the inlet and outlet pump cannulae without distortion to their normal shape, thus avoiding flow disturbance and consequent formation of blood clots.
